# A New Efficient Trocar to Insert the Wound Drainage Tube: Jackson–Pratt Drainage Tube

**DOI:** 10.1089/vid.2016.0065

**Published:** 2017-02-13

**Authors:** Yu Seob Shin, Jae Hyung You, Myoung-Hwan Ko, Jong Kwan Park

**Affiliations:** 1 Department of Urology, Chonbuk National University and Research Institute of Clinical Medicine of Chonbuk National University-Biomedical Research Institute and Clinical Trial Center of Medical Device of Chonbuk National University Hospital, Jeonju, Korea.; 2 Yu Seob Shin and Jae Hyung You contributed equally to this work.; 3 Department of Urology, Chonbuk National University and Research Institute of Clinical Medicine of Chonbuk National University-Biomedical Research Institute and Clinical Trial Center of Medical Device of Chonbuk National University Hospital, Jeonju, Korea.; 4 Yu Seob Shin and Jae Hyung You contributed equally to this work.; Department of Physical Medicine and Rehabilitation, Chonbuk National University and Research Institute of Clinical Medicine of Chonbuk National University-Biomedical Research Institute and Clinical Trial Center of Medical Device of Chonbuk National University Hospital, Jeonju, Korea.; Department of Urology, Chonbuk National University and Research Institute of Clinical Medicine of Chonbuk National University-Biomedical Research Institute and Clinical Trial Center of Medical Device of Chonbuk National University Hospital, Jeonju, Korea.

**Keywords:** trocar, insert, wound drainage tube, brand new, quadrangle grip

## Abstract

***Introduction:*** A trocar is used to pierce the muscle and skin to insert the wound drainage tube into the operating field to drain excess blood and fluid just after the operation.^1^ However, the handle of the trocar always becomes slippery, making it difficult for the operator to control it. Sometimes, the lack of control of a trocar may lead the trocar tip to protrude forward in an unwanted direction and penetrate into another organ of the patient. Sometimes, the operator could hurt his hand while pulling the trocar out by the sharp edge of trocar tip. In this video clip, we would like to introduce a brand new trocar that can be used to insert the wound drainage tube.

***Materials and Methods:*** The new trocar has two quadrangle grips (SUNGWON MEDICAL CO., LTD., Korea Patent; 10-1540199). One of the grips has an embossing surface to prevent the trocar from rotating unnecessarily when the operator pushes the trocar to penetrate the muscle and skin. The upper side of the grip has a sunken surface that helps the operator to direct the trocar tip using tactile sensation in the absence of visibility. The other grip has a smooth surface near the trocar tip to prevent it from slipping when the operator picks, by hand, the trocar that has emerged out of the skin. We measured the roughness and friction of the hand grip of the trocars by using Optical 3D Surface Measurement System and scanning of electron microscope. In addition, we assessed the questionnaires answered by surgeons on three domains, including the unwanted turn of the trocar tip, the unwanted sliding of the trocar grip, and the directivity of the trocar tip. The three domains were calculated by the amount of degree that a surgeon felt ranging across a continuum from 0 to an extreme amount of 10 subscales.

***Results:*** This trocar is routinely used for all open procedures. The roughness and friction of the brand new trocar had higher roughness and friction than the old trocar (roughness: 0.4365 ± 0.2664 and 1.9988 ± 0.72783 Ra, *p* < 0.05; friction: 3.059 ± 0.286 and 3.486 ± 0.428 N, *p* < 0.05). The questionnaire was attempted by 32 surgeons. The score of the unwanted turn of the previous trocar tip was higher than that of the brand new trocar (6.46 ± 3.09 *vs* 0.65 ± 1.03, *p* < 0.001). The score of the unwanted sliding of trocar grip of the previous trocar was higher than that of the new trocar (7.46 ± 1.98 *vs* 1.50 ± 1.90, *p* < 0.001). The score of the directivity of the trocar tip of the new trocar was higher than that of the old trocar (8.93 ± 1.47 *vs* 3.09 ± 2.30, *p* < 0.001).

***Conclusion*:** Trocar insertion using the new trocar that has quadrangle grips with sanding surface is an efficient and feasible technique for patients and surgeons.

**Acknowledgments:** The authors thank the members of the medical device clinical trial center of Chonbuk National University Hospital for helpful discussions. This research was also supported by a grant of the Korea Health Technology R&D Project through the Korea Health Industry Development Institute (KHIDI), funded by the Ministry of Health & Welfare, Republic of Korea (Grant No.: HI15C1529). However, the Ministry of Health & Welfare, Republic of Korea, had no role in design or conduct of the study, including collection, management, analysis, or interpretation of the data in addition to preparation, review, or approval of the article.

*J.K.P. has patent of this trocar and the patent was transferred to SUNGWON MEDICAL device company. The other authors have nothing to disclose.* *SUNGWON MEDICAL device company had no role in design or conduct of the study, including collection, management, analysis, or interpretation of the data in addition to preparation, review, or approval of the article.*

Runtime of video: 3 mins

## Graphical abstract

**Figure:**
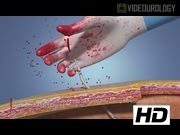

**Figure:**
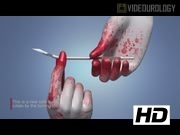

**Figure:**
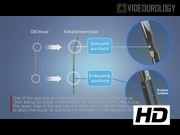

## Video

## Files

**PDF:** 

**QT:** 

**HD:** 
